# Effect of Serum Netrin-1 Levels on Diagnosis and Prognosis in Patients Admitted to the Emergency Service for Acute Coronary Syndrome

**DOI:** 10.7759/cureus.7741

**Published:** 2020-04-20

**Authors:** Huseyin Mutlu, Nazire Akilli, Basar Cander, Öznur Köylü, Murat Gul, Ramazan Köylü

**Affiliations:** 1 Emergency Medicine, Faculty of Medicine, Aksaray University, Aksaray, TUR; 2 Emergency Medicine, Konya Training and Research Hospital, University of Health Sciences, Konya, TUR; 3 Emergency Medicine, Kanuni Sultan Süleyman Training and Research Hospital, University of Health Sciences, Istanbul, TUR; 4 Biochemistry, Konya Training and Research Hospital, University of Health Sciences, Konya, TUR; 5 Cardiology, Aksaray University, Faculty of Medicine, Aksaray, TUR

**Keywords:** netrin-1, acute coronary syndrome, reperfusion

## Abstract

Background

Netrin-1 is a recently discovered diagnostic biomarker that indicates atherosclerosis, angiogenesis, and ischemia-reperfusion damage. There are no human studies about Netrin-1 in acute coronary syndrome (ACS). The purpose of the present study was to investigate Netrin-1 levels in the early diagnosis and successful reperfusion of ACS.

Method

The study was conducted with 188 patients diagnosed with ACS and 50 healthy subjects at the emergency unit in a prospective design. Blood samples were collected from the patient group at initial admission and after angiography. The control group consisted of healthy adult subjects without any disease. Netrin-1 levels were studied in both groups.

Results

The Netrin-1 levels of the patient group at the time of admission were found to be higher than of the control group (p<0.001). In the patient group, netrin-1 levels measured at initial admission (1.53±0.19) and after angiography (1.49±0.19) were determined to be statistically significant (p:0.049). In the patient group, where the Thrombolysis in Myocardial Infarction (TIMI) 3 flow was established after angiography, netrin-1 levels were detected to be low (p:0.039). Netrin-1 levels obtained at the time of admission were determined to be significantly different in the Global Registry of Acute Coronary Events (GRACE) moderate and high-risk groups in comparison to the low-risk group (p:0.017).

Conclusion

Netrin-1 was shown to increase in the early diagnosis of ACS and to decrease in patients for whom reperfusion was established after angiography. Therefore, Netrin-1 can be an important biomarker as an indicator of diagnosis and successful reperfusion in ACS.

## Introduction

In the USA and Europe, approximately 15-20 million individuals present to the emergency rooms with acute chest pain and other symptoms of acute coronary syndrome (ACS) annually. One-third of these visits are associated with ACS [1-3]. Two components of ACS, acute myocardial infarction and unstable angina, are the major causes of death and disability worldwide [3-4]. In ACS, the risk of mortality and the benefit of early revascularization are the highest in the initial hours; therefore, early diagnosis is crucial [1-3]. Procedures for revascularization do not guarantee myocardial perfusion. Therefore, various markers are evaluated to demonstrate the success of reperfusion and reduced ischemia [5-6]. It has been shown that clinical risk scores, such as Global Registry of Acute Coronary Events (GRACE) and Thrombolysis in Myocardial Ischemia (TIMI), which have significant distinguishing power in calculating the hospital mortality risks, hospital discharge, and six-month mortality risks mentioned in ACS guidelines, can be improved with some biomarkers and new parameters [7-8].

Because of insufficient early diagnosis in ACS, several biomarker studies have been carried out in order to determine the success of reperfusion after angiography and to improve clinical risk scores. Some of these biomarkers are copeptin, mid-regional pro-adrenomedullin, B-type natriuretic peptide, ischemia-modified albumin (IMA), heart-type fatty acid-binding protein, thymosin beta 4, and amyloid-β (1-40) (Aβ40) [5-7]. There are also ongoing studies aiming to identify novel biomarkers [5-8].

Netrins are important precursor proteins for neural and vascular development [9]. Animal studies have shown that Netrin-1 is an important cardioprotective agent in atherosclerosis, angiogenesis, and ischemia-reperfusion injury [10]. Other animal studies have shown that Netrin-1 protects the heart against myocardial ischemia-reperfusion injury and reduces the size of the infarction in the heart by increasing nitric oxide (NO) [11]. Nguyen and Cai have shown that Netrin-1 induces angiogenesis in the endothelium by increasing NO release [12].

These data indicate that Netrin-1 can be a diagnostic marker and an indicator of reperfusion in ACS. Our primary aim is to compare the Netrin-1 levels of patients with ACS with healthy controls, and our secondary aim is to assess the alterations in Netrin-1 levels of patients with ACS after angiography.

## Materials and methods

Subjects and study design

This study was approved by the Necmettin Erbakan University Meram Faculty of Medicine Clinical Research Ethics Advisory Board's decision, dated October 23, 2013, numbered 2013-23, and performed in Konya Training and Research Hospital Emergency Medicine Clinic. The study was designed as a controlled, open, observational prospective clinical trial. Prior to the study, informed consent was obtained from each participant in accordance with the Helsinki Declaration of the World Medical Association.

Patients over the age of 18 years who were diagnosed with ACS, scheduled for angiography, and accepted to undergo angiography were included in the study.

Exclusion criteria

The exclusion criteria included:

Patients with a history of a cerebrovascular event;

Patients with a neurological disease;

Pregnant and lactating women;

Patients with liver failure;

Patients with a malignancy;

Patients using anticonvulsant and nephrotoxic drugs;

Patients with chronic kidney disease;

Patients who did not undergo angiography; and

Patients who developed acute renal failure after angiography.

**Figure 1:**
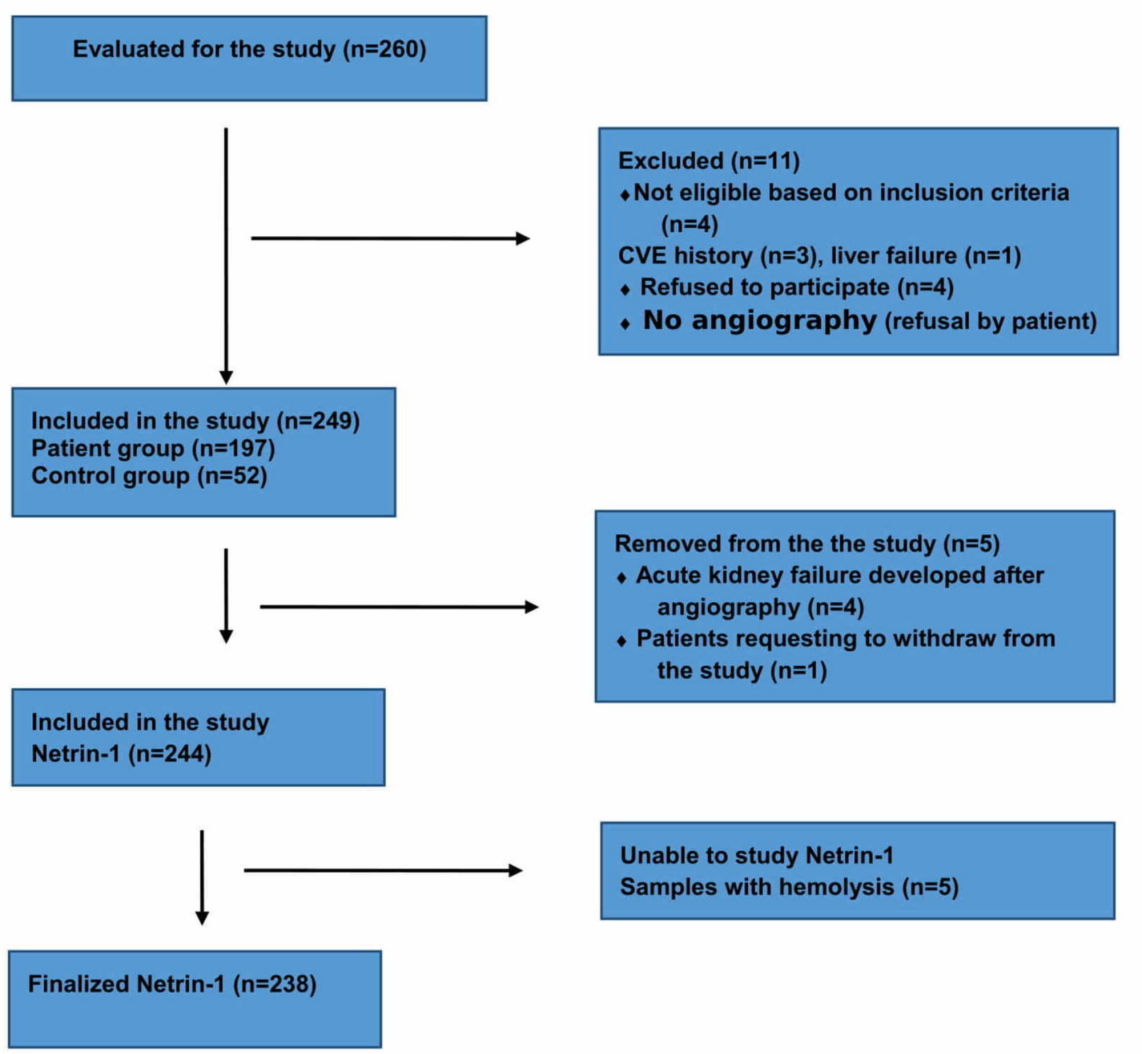
Flow diagram of the study

Patients who did not want to participate in the study were excluded from the study. A total of 197 patients and 52 healthy controls were included in the study. A flow diagram of the study can be seen in Figure 1.

The demographic characteristics, patient history, vital signs, laboratory findings, coronary angiography (CAG) findings, and echocardiography findings of the patients were recorded. Netrin-1 levels were analyzed with blood samples taken at presentation and six to eight hours after CAG. CAG results were evaluated with TIMI flow criteria. The antegrade radiocontrast flow of the infarct-related artery was determined on the final coronary angiogram by the operator using TIMI criteria. The TIMI flow grades have been defined previously. In brief, grade 0 perfusion is no antegrade flow beyond the point of occlusion; grade 1 is minimal incomplete perfusion of contrast medium around the clot; grade 2 (partial perfusion) is complete but delayed perfusion of the distal coronary bed with contrast material; and grade 3 (complete perfusion) is an antegrade flow to the entire distal bed at a normal rate [13]. The patients were divided into two groups as those with and without TIMI 3 flow and their Netrin-1 levels were compared.

To evaluate clinical risk factors, all patients were divided into two groups as ACS patients with ST-elevation (STEMI) and without ST elevation (NSTEMI) and unstable angina pectoris (UAP). Criteria for STEMI patients were as follows: a) Typical resting chest pain and/or ischemic symptoms persisting longer than 20 min; b) ≥0.1 mV ST elevation in all leads except V2-V3 from point J on two consecutive leads in ECG and ≥0.2 mV ST elevation in V2-V3 and new ST-T change or newly formed left bundle branch block; c) positive markers showing myocardial necrosis. Criteria for NSTEMI patients were as follows: a) no ST-segment elevation as previously described and b) positive markers showing myocardial necrosis. Criteria for UAP were as follows: a) absence of 1 mm or more ST-segment elevation as defined, b) negative markers showing myocardial necrosis and the presence of angina pectoris in any of the following three features: 1) angina occurring at rest or over a long period of time (usually >20 min), new-onset angina of at least class III severity according to the Canadian Cardiovascular Society (CCS) classification, a recent progression of at least one CCS class in pain classification, or a new angina acceleration of at least CCS class III [14-15].

GRACE and TIMI clinical risk assessments were performed in patients. In the GRACE scoring system, risk scores are calculated using computer programs with the help of age, heart rate, systolic blood pressure, Killip class, electrocardiogram (ECG) and cardiac marker changes, creatinine level, and cardiac arrest history variables. Patients were divided into three groups according to these scores as low (108), moderate (109-140), and high (141-372) risk groups, and their Netrin-1 values were compared. TIMI risk scores were calculated for STEMI patients using age, weight, time of treatment initiation, heart rate, systolic blood pressure, Killip class, ECG changes, and diabetes mellitus (DM), hypertension (HT), or angina history. Patients were divided into two groups according to these scores as low-risk and high-risk groups, and their Netrin-1 values were compared. TIMI risk scores were calculated for UA/NSTEMI patients using age ≥65 years, ≥3 risk factors for coronary artery disease (CAD) (risk factors included family history of CAD, HT, hypercholesterolemia, DM, or being a current smoker), known CAD (stenosis ≥ 50%), acetylsalicylic acid (ASA) use in past seven days, severe angina (≥2 episodes within 24 h), ST changes ≥0.5 mm, and positive cardiac marker. Patients were divided into two groups as low- and high-risk groups and their Netrin-1 values ​​were compared [16-17].

Netrin-1 quantification in plasma

The antecubital area on the front side of the elbow was cleaned with 70% alcohol; blood samples were then collected from that area and placed into ethylenediaminetetraacetic acid (EDTA) tubes. After centrifugation at 4°C at 2800 rpm for 20 min, plasma samples were placed in Eppendorf tubes and stored at −80°C until the time of analysis.

The plasma Netrin-1 level was measured by enzyme-linked immunosorbent assay (MyBioSource MBS044526; MyBioSource, Inc., San Diego, California). All assays were performed in duplicate. Plasma Netrin-1 is expressed in picograms (pg) per mg.

Statistical analysis

The SPSS for Windows 15.0 program (SPSS Inc., Chicago, Illinois) was used for the statistical analysis of data. When comparing the patient and control groups, the chi-square or Fisher’s exact test was used for categorical variables and the independent samples t-test was used for normally distributed variables. The laboratory parameters of the patients were compared with the dependent samples student’s t-test for normally distributed variables and the Wilcoxon test for non-normally distributed variables. The relationship between the laboratory parameters of the groups formed according to the clinical risk classification results was evaluated with the one-way analysis of variance (ANOVA) test. Correlations were analyzed by Pearson’s correlation test. P-values below 0.05 were considered statistically significant.

## Results

A total of 238 individuals - 188 patients and 50 controls - were included in the study. The mean age was 63.8 ± 14.0 years in the patient group, 65.9 ± 17.8 years in the control group, and there was no statistical difference (p = 0.867). The demographic characteristics of the patient and control groups are shown in Table 1.

**Table 1:** Demographic characteristics of the patients Data are expressed as mean ± standard deviation (SD), number (percentage), or median (interquartile range; IQR) NSTEMI, non-ST segment elevation myocardial infarction; STEMI, ST-segment elevation myocardial infarction; GRACE: Global Registry of Acute Coronary Events

Variables	Patients group (n:188)	Control group (n:50)	p-value
Age (years) Mean±SD	63.8±14.0	65.9±17.8	0.177
Sex (%)			
Female	64 (36.0%)	20(40.0%)	0.353
Male	124 (64.0%)	30 (60.0%)	0.678
Systolic blood pressure (mmHg)	121.5 (30)	125,3(29)	0.364
Diastolic blood pressure (mmHg)	70 (10)	68.6(11)	0.545
Heart rate (beats/min)	79.8±14.1	81.7±13.7	0,257
Admission Netrin-1(pg/ml)	1.53±0.19	1.38±0.18	<0.001
Netrin-1 at 4th hour after angiography (pg/ml)	1.49±0.19		
Diabetes mellitus	69 (36.7%)	15(30%)	0,084
Hypertension	99 (52.7%)	24(48%)	0,098
Previous myocardial infarction	53 (28.2%)	8(16%)	<0.001
Hypercholesterolemia	27 (14.4%)	6(12%)	0,224
Family history of coronary artery disease	85 (45.1%)	19(38%)	0,146
Aspirin	51 (27.1%)	18(36)	<0.001
Current smoker	83 (44.1%)	23(46%)	0,482
Clinical presentation STEMI	82 (43.6%)		
NSTEMI	106 (56.4%)		
GRACE	133.15±36.26		
GRACE risk group	49 (26.1%)		
Low	65 (34.6%)		
Moderate	74 (39.4%)		
High	4.44±6.79		

Netrin-1 levels were statistically higher in the patient group as compared with the control group (p < 0.001) (Table 1).

Netrin-1 levels after angiography were lower in the patient group with TIMI 3 flow as compared with Netrin-1 levels at presentation (p < 0.001). In the patient group without TIMI 3 flow, there was no difference between Netrin-1 levels after angiography and at presentation (p = 0.37) (Table 2).

**Table 2:** Netrin-1 levels based on TIMI-3 TIMI: Thrombolysis in Myocardial Ischemia

	Admission Netrin-1 value	After angiography Netrin-1 value	p-value
With TIMI-3 flow n:104	1.56±0.18	1.46±0.20	<0.001
Without TIMI-3 flow n:84	1.49±0.20	1.52±0.17	0.37

In NSTEMI patients according to TIMI, Netrin-1 levels at the time of presentation were higher in the high-risk group, and the difference was almost statistically significant (p = 0.07).

There was no correlation between Netrin-1 levels at admission and after angiography, and ejection fraction (EF), troponin, and CK-MB. Netrin-1 values at presentation were correlated with NSTEMI TIMI and GRACE (r: 0.3, r: 0.2, respectively, p < 0.05).

Netrin-1 levels at presentation were found to be significantly different in the GRACE medium and high-risk groups as compared with the low-risk group (p= 0.017). Post-hoc analysis revealed that this was due to the difference between Group 1 and Group 2 and between Group 1 and Group 3. There was no significant difference between Group 2 and Group 3 (p = 0.895) (Table 3).

**Table 3:** GRACE risk group and Netrin-1 levels a Significantly different when compared against moderate-risk group p:0.038 b Significantly different when compared against high-risk group p:0.023 GRACE: Global Registry of Acute Coronary Events, Netrin-1 (picogm/ml)

	Low risk n:49	Moderate risk n:65	High risk n:74	p-value
Admission Netrin-1 value	1.46 (0.18)^a,b^	1.55 (0.20)	1.56 (0.18)	0.017
After Angiography Netrin-1 value	1.50 (0.19)	1.50 (0.19)	1.49 (0.20)	0.877

## Discussion

In the present study, we found that Netrin-1 increased at the time of presentation in patients with ACS, decreased after angiography in patients with TIMI 3 flow, and was higher in high-risk groups according to clinical risk classifications such as TIMI and GRACE.

Netrins are important precursor proteins for neural and vascular development [9]. Five types of Netrins have been identified in mammals (1, 3, 4, G1, and G2). It is one of the extracellular proteins that direct cell and axon migration during embryogenesis. Netrin-1 stimulates proliferation, induces migration, and promotes the adhesion of endothelial cells and vascular smooth muscle cells through the specific activity of vascular endothelial growth factor and platelet-derived growth factor, respectively [18]. It is an important cardioprotective agent in angiogenesis and ischemia-reperfusion injury [10]. Netrin-1 has been shown to protect against a myocardial ischemia-reperfusion injury and to reduce infarction size by inhibiting apoptosis and increasing myositis function through ERK1/2 inactivation immediately followed by NO production from serine 1177-phosphorylated eNOS in cardiac myocytes [10].

Nguyen and Cai showed that Netrin-1 increased NO release and induced angiogenesis by activating growth and migration in endothelial cells [12]. In an animal study, Durrani et al. showed that synthesized Netrin-1 decreased apoptosis in endothelial cells and increased vascular density to decrease ischemia-reperfusion injury [18]. These studies suggest that Netrin-1 levels may be increased in ACS [19]. In support of these studies, our results revealed that Netrin-1 levels were higher in ACS patients as compared with healthy controls at the time of presentation, which indicates that Netrin-1 may act as a guide in the early diagnosis of ACS.

In the study of Hjortshøj et al., Netrin-1 was shown to protect the heart from ischemic injury in mice [20]. Other animal studies have shown that Netrin-1 induces angiogenesis, protects against a myocardial ischemia-reperfusion injury, and decreases infarction size by increasing NO. In addition, it has been shown that the release of Netrin-1 and NO decreases with decreasing ischemia [12,18]. These studies show that Netrin-1 may be an indicator of the success of post-angiography reperfusion and decreased ischemia in ACS such as IMA, endocan, and thymosin beta 4 [6,21-22]. Based on these studies, we assumed that the Netrin-1 level would be increased at the time of presentation to the hospital in ACS and the Netrin-1 level would decrease after achieving flow following angiography. Netrin-1 levels after angiography were found to be significantly lower in the patient group with TIMI 3 flow after angiography, which showed us that Netrin-1 may be a reperfusion marker in ACS.

Mohammad et al. found that the Netrin-1 level increased in acute renal failure (ARF) induced by ischemia, and Netrin-1 decreased with the improvement of ARF [23]. Mirakaj et al. showed that Netrin-1 increased in acute lung injury [24]. Guo et al. showed that Netrin-1 increased and was a prognostic marker in ischemic stroke [25]. These studies indicate that Netrin-1 would be increased in ischemic conditions. In support of these studies, our results showed that Netrin-1 levels were higher in patients with ACS as compared to the control group.

The studies of Stamatelopoulos et al. on amyloid-β (1-40) and Li et al. on serum S100A1 found that these biomarkers may help the prognosis in clinical risk scoring, such as GRACE and TIMI, which are defined as mortality predictors in ACS guidelines [7-8]. In the present study, Netrin-1 levels measured at the time of presentation to the hospital were found to be significantly different in the medium and high-risk GRACE groups as compared with the low-risk group (p = 0.017). It was observed that this difference was caused by the difference between the low- and medium- and low- and high-risk groups. In NSTEMI patients, Netrin-1 levels at presentation to the hospital were higher in the high-risk group as compared with the low-risk group according to TIMI scoring, which was close to statistical significance (p = 0.07). These results show that Netrin-1 is a biomarker with the potential to assist TIMI and GRACE clinical risk scoring and prognosis.

In the study of Aragam et al., in-hospital mortality in ACS was 4% (25). In the present study, the in-hospital mortality rate was 5.3%, and no relationship was found between Netrin-1 levels and mortality (p > 0.05).

Netrin-1 can be used in the early diagnosis of ACS, as an indicator of reperfusion, and to predict prognosis with clinical risk scoring. In addition, we believe that Netrin-1 is a candidate marker for further study in thrombotic-ischemic events, such as cerebrovascular events, peripheral artery disease, pulmonary embolism, and mesenteric ischemia, wherein angiogenesis increases.

We evaluated the effectiveness of only one biomarker and believe that other biomarkers that have diagnostic and prognostic value should be used together with Netrin-1. However, this study will shed light on other studies, as it is the first such study in the literature. We believe that patients should be followed up for six months in terms of mortality.

## Conclusions

It is evident that there is a need for new biomarkers in ACS. Many biomarkers are used in the literature to determine diagnosis, prognosis, mortality, and reperfusion in ACS. In addition, many biomarkers are being tried in the clinical risk scoring used in ACS. However, the reliability of these markers is controversial. Based on the findings obtained in the present study and in previous studies on similar subjects, we found that serum Netrin-1 levels in patients with ACS who present to the emergency department may have a diagnostic value, be an indicator of reperfusion, or be a novel risk factor in clinical risk scores. However, it is clear that further studies are needed on this subject.
